# Brain gangliosides of a transgenic mouse model of Alzheimer's disease with deficiency in GD3-synthase: expression of elevated levels of a cholinergic-specific ganglioside, GT1aα

**DOI:** 10.1042/AN20130006

**Published:** 2013-05-30

**Authors:** Toshio Ariga, Yutaka Itokazu, Michael P. McDonald, Yoshio Hirabayashi, Susumu Ando, Robert K. Yu

**Affiliations:** *Institute of Molecular Medicine and Genetics and Institute of Neuroscience, Medical College of Georgia, Georgia Regents University, Augusta, GA 30912, and VA Medical Center, Augusta, GA 30904, U.S.A.; †Departments of Neurology and Anatomy and Neurobiology, University of Tennessee Health Sciences Center, Memphis, TN, U.S.A.; ‡Laboratory for Molecular Neuroscience, Brain Science Institute, Saitama, Japan; §Department of Biochemistry, The Tokyo Metropolitan Institute of Gerontology, Itabashi-ku, Tokyo, Japan

**Keywords:** Alzheimer’s disease, amyloid β protein, Chol-1α ganglioside, transgenic mouse, Aβ, amyloid β-protein, AD, Alzheimer’s disease, APP, amyloid precursor protein, HPTLC, high-performance TLC, PSEN, presenilin, Tg, transgenic, WT, wild-type, GD3S or STII, GD3-synthase, mAb, monoclonal antibody, NeuNAc, *N*-acetylneuraminic acid or sialic acid

## Abstract

In order to examine the potential involvement of gangliosides in AD (Alzheimer's disease), we compared the ganglioside compositions of the brains of a double-transgenic (Tg) mouse model [APP (amyloid precursor protein)/PSEN1 (presenilin)] of AD and a triple mutant mouse model with an additional deletion of the *GD3S* (GD3-synthase) gene (APP/PSEN1/GD3S^−/−^). These animals were chosen since it was previously reported that APP/PSEN1/GD3S^−/−^ triple-mutant mice performed as well as WT (wild-type) control and GD3S^−/−^ mice on a number of reference memory tasks. Cholinergic neuron-specific gangliosides, such as GT1aα and GQ1bα, were elevated in the brains of double-Tg mice (APP/PSEN1), as compared with those of WT mice. Remarkably, in the triple mutant mouse brains (APP/PSEN1/GD3S^−/−^), the concentration of GT1aα was elevated and as expected there was no expression of GQ1bα. On the other hand, the level of c-series gangliosides, including GT3, was significantly reduced in the double-Tg mouse brain as compared with the WT. Thus, the disruption of the gene of a specific ganglioside-synthase, GD3S, altered the expression of cholinergic neuron-specific gangliosides. Our data thus suggest the intriguing possibility that the elevated cholinergic-specific ganglioside, GT1aα, in the triple mutant mouse brains (APP/PSEN1/GD3S^−/−^) may contribute to the memory retention in these mice.

## INTRODUCTION

Gangliosides are sialic acid-containing GSLs (glycosphingolipids) expressed primarily, but not exclusively, in the outer leaflet of the plasma membrane of cells of all vertebrates [gangliosides are abbreviated using the nomenclature rules of IUPAC-IUB (1977) and according to Svennerholm (1963)]. Those GSLs are particularly abundant in the nervous system. Ganglioside metabolism is closely associated with brain development, and in some cases with the pathology of neurodegenerative diseases such as AD (Alzheimer's disease). AD is the most common type of dementia with clinical symptoms that include deficits in memory, judgment, thinking and behaviour. The accumulation of Aβs (amyloid β-proteins) is one of the major pathological hallmarks in AD. Although the functions of gangliosides in the pathogenic mechanisms of AD are not completely understood, evidence has accumulated to indicate a critical role for gangliosides in binding to and aggregation of Aβs, the toxic peptides in AD brain. Thus, gangliosides are frequently used as biomarkers associated with the pathological events of AD (Ariga et al., 2011; Yanagisawa, 2011).

We recently examined the ganglioside composition of the brains of a double-Tg (transgenic) mouse model [APP (amyloid precursor protein)/PSEN1 (presenilin-1)] of AD that co-expresses mouse/human chimeric APP with the Swedish double-mutation and human PSEN1 with a deletion of exon 9 (Ariga et al., 2010). Although the accumulation of Aβ was confirmed in the double-Tg mouse brains and sera, the content and composition of the major ganglio-N-tetraosyl-series gangliosides in the brains were not significantly different between the double-Tg mice and WT (wild-type) mice (Ariga et al., 2010). This latter finding was in line with that reported by Sawamura et al. (2000), who also did not detect any changes in the major ganglioside patterns in the mutant PSEN2 double-Tg mice as compared with those in WT mice. The most interesting finding of our previous study was the increased expression of the Chol-1α antigens, GT1aα and GQ1bα, especially GQ1bα, in the brains of double-Tg (APP/PSEN1) mice as compared with those in WT mice.

A2B5 antigens, including the c-series gangliosides such as GT3, GT1c and GQ1c, are well-known marker molecules of glial precursor cells, O-2A progenitor cells, which differentiate into type-2 astrocytes and oligodendrocytes (Raff et al., 1983; Zhang, 2001). GT3 was detected in the developing embryonic mouse brains using A2B5 mAb (monoclonal antibody) and the expression of GT3 was significantly diminished during later development (Ngamukote et al., 2007).

In a previous study, we established the triple-Tg mouse (APP/PSEN1/GD3S^−/−^) by cross-breeding the double-Tg (APP/PSEN1) mouse model of AD with GD3S^−/−^ mice (Bernardo et al., 2009). GD3S (GD3-synthase) is responsible for the biosynthesis of GD3, a key intermediate for the biosynthesis of b- and c-series gangliosides, including two of the major brain gangliosides: GD1b and GT1b. Surprisingly, Aβ plaques and the associated neuropathology were almost completely absent in the triple-Tg (APP/PSEN1/GD3S^−/−^) mice, resulting in cognitive improvement. These triple-Tg mice performed as well as WT controls on all behavioural tests of sensorimotor function, anxiety and cognition. We reasoned that there must be a correlation between the pathogenesis of AD and the unique ganglioside composition in the GD3S knockout animals. For this reason, we undertook a detailed analysis of the minor gangliosides, particularly GT1aα, GQ1bα and GT3, in the brain of the double-Tg and triple-Tg mice and found that these minor gangliosides were significantly altered. The elevated expression of GT1aα in the triple-Tg animals was particularly pronounced. Our data suggest that the elevated cholinergic-specific ganglioside, GT1aα, in the triple-mutant mouse brains (APP/PSEN1/GD3S^−/−^) may contribute to diminished plaque formation and, concomitantly, functional recovery in these mice.

## MATERIALS AND METHODS

### Gangliosides and antibodies

Chol-1α gangliosides, GQ1bα and GT1bα, were isolated from bovine brains (Ando et al., 1992; Hirabayashi et al., 1992). GD3 ganglioside was isolated from buttermilk (Ren et al., 1992). A mouse mAb, A2B5, was originally derived from a mouse immunized with 8-day chicken embryo retinas (Eisenbarth et al., 1979); the hybridoma cells were obtained from ATCC (Manassas). Mouse Chol-1α mAb (GGR-41) was obtained by immunizing mice with a GQ1bα-enriched ganglioside fraction extracted from bovine brain according to conventional procedures (Kusunoki et al., 1993).

### Animals

The double-Tg mouse model of AD (APP_swe_+PSEN1) was purchased from the Jackson Laboratory (Bar Harbor; stock #004462) and propagated in our laboratories. The mice express the chimeric mouse/human APP gene harbouring the Swedish double-mutation (K595N/M596L) and a human PSEN1 gene with a deletion of exon 9. The triple-Tg mouse model of AD (APP_swe_+PSEN1 lacking the gene encoding GD3S (ST-II; ST8 α-N-acetyl-neuraminide, α-2,8-sialyltransferase 1; *St8sia1*) was prepared as previously described (Bernardo et al., 2009). All animals, both male and female, used for ganglioside analysis were from 7 to 9 months of age. The use of animals for this study was approved by the Institutional Animal Care and Use Committees at the Georgia Regents University and University of Tennessee Health Sciences Center.

### Ganglioside isolation

Gangliosides were isolated from brain slices containing hippocampal/cortical tissue from WT, GD3S^−/−^, double-Tg and triple-Tg mice (*n*=3 per group) as previously described (Yu and Ledeen, 1972) with some modifications (Ariga et al., 1988). Briefly, total lipids were extracted from brain tissues with 5 volumes each of chloroform–methanol (1:2, 1:1 and 2:1, v/v) and chloroform–methanol–water (30:60:8, v/v; Solvent A). Then, the combined extracts were evaporated and dissolved in 5 ml of Solvent A, and applied to a DEAE-Sephadex A-25 column (acetate form, 2-ml bed volume), followed by elution with 20 ml of solvent A to remove the neutral lipids. The acidic lipid fraction, containing gangliosides, was then eluted with 20 ml of chloroform–methanol–0.8 M sodium acetate in water (30:60:8, v/v, Solvent B), followed by desalting using Sep-Pak Cartridge column chromatography (Waters Assoc.) (Kubo and Hoshi, 1985). The lipid-bound sialic acid [NeuNAc (*N*-acetylneuraminic acid or sialic acid)] content in the acidic lipid fraction was determined by the resorcinol–HCl reagent (Svennerholm, 1957). A portion of the acidic lipid fraction, equivalent to 1.5 μg of NeuNAc, was applied to an HPTLC (high-performance thin-layer chromatography) plate and developed with the solvent system of chloroform–methanol–water containing 0.2% CaCl_2_·H_2_O (50:45:10, v/v). Ganglioside bands were visualized by spraying with the resorcinol–HCl reagent followed by heating at 100°C.

### HPTLC-immunostaining using anti-Chol-1α and A2B5 mAb

After developing the HPTLC plate described above, the plate was coated in a solution of *n*-hexane containing 0.4% (w/v) polyisobutylmethacrylate (Sigma Co.) for 1 min. After drying, the plate was incubated with anti-Chol-1α mAb [GGR-41; IgG (immunoglobulin G)] or A2B5 mAb (IgM) diluted with 1% (w/v) BSA in PBS at 4°C overnight. The plate was then incubated with an HRP (horse radish peroxidase)-conjugated IgG or IgM secondary antibody (Jackson ImmunoResearch) diluted with 1% (w/v) BSA in PBS for 1 h. Bands were detected using the Western Lightning Western blot chemiluminescence reagent (Perkin Elmer Life and Analytical Sciences). After the plate was dipped in chloroform to remove excess polymer, ganglioside bands were visualized by spraying with the orcinol–sulfuric acid reagent at 100°C.

## RESULTS AND DISCUSSION

**Figure 1:**
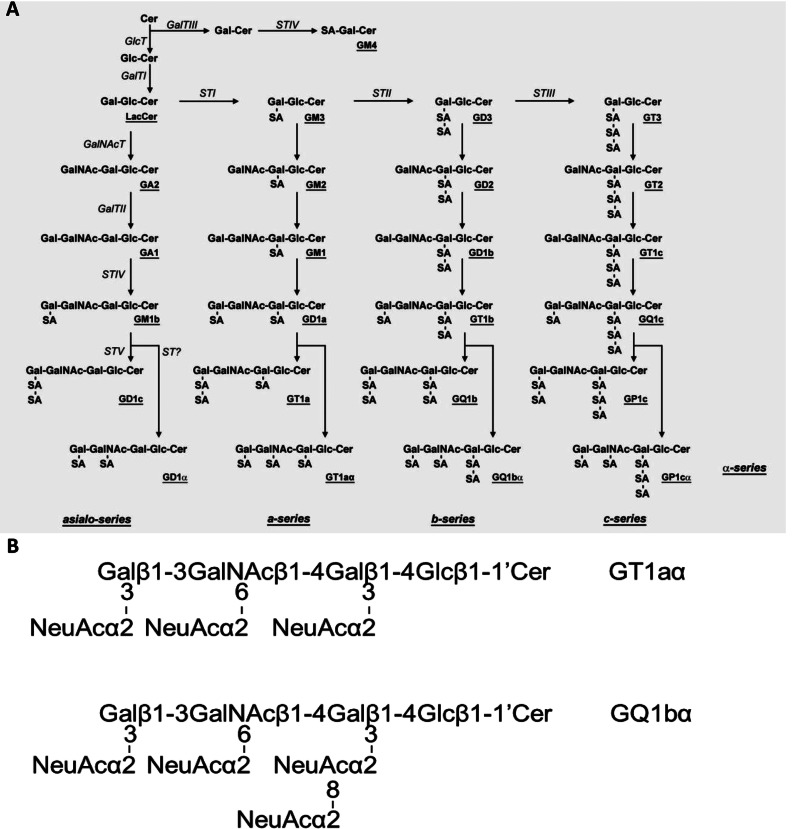
Structure and biosynthetic pathways of brain gangliosides The nomenclature of gangliosides and other glycolipids are based on that of Svennerholm (1963) and the IUPAC-IUBMB Joint Commission on Biochemical Nomenclature (1977). (**A**) The glycosyltransferases catalysing the synthesis of each of the glycosphingolipids, including gangliosides, are italicized. GD1aα, GT1aα, GQ1bα and GP1cα are classified as belonging to the α-series gangliosides. (**B**) Structure of Chol-1α gangliosides: GT1aα, GQ1bα.

**Figure 2:**
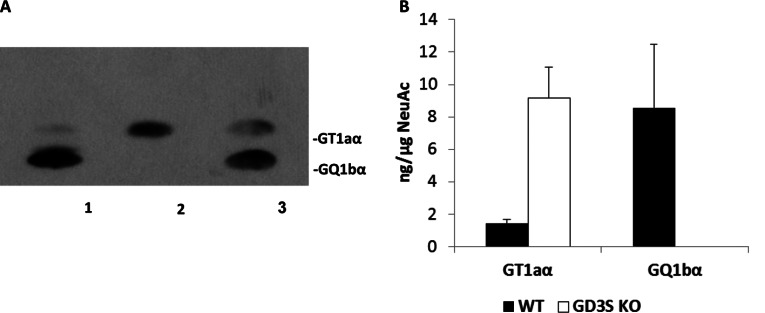
HPTLC-immunostaining analysis of the expression of Chol-1α antigens in the brains of WT and GD3S^−/−^ mice Gangliosides were separated by HPTLC with the solvent system of chloroform/methanol/0.2% CaCl_2_ (50:45:10, by volume) and bands were visualized by immunostaining with anti-Chol-1α mAb (GGR-41) as described in the text (**A**). Each lane contained 3 μg of total lipid-bound sialic acid, and only the immunoreactive bands are shown. Lane 1, Gangliosides in the brain of WT mice; lane 2, gangliosides in the brain of GD3S knockout mice; lane 3, authentic GT1aα (20 ng) and GQ1bα (20 ng), respectively. (**B**) shows the concentrations of GT1aα and GQ1bα as determined by densitometric scanning of the intensities shown in (**A**). The *y*-axis represents ng (sialic acid) of the immunoreactive ganglioside in the total ganglioside fraction. The bar denotes S.E.M. of three independent determinations (*n*=3).

**Figure 3:**
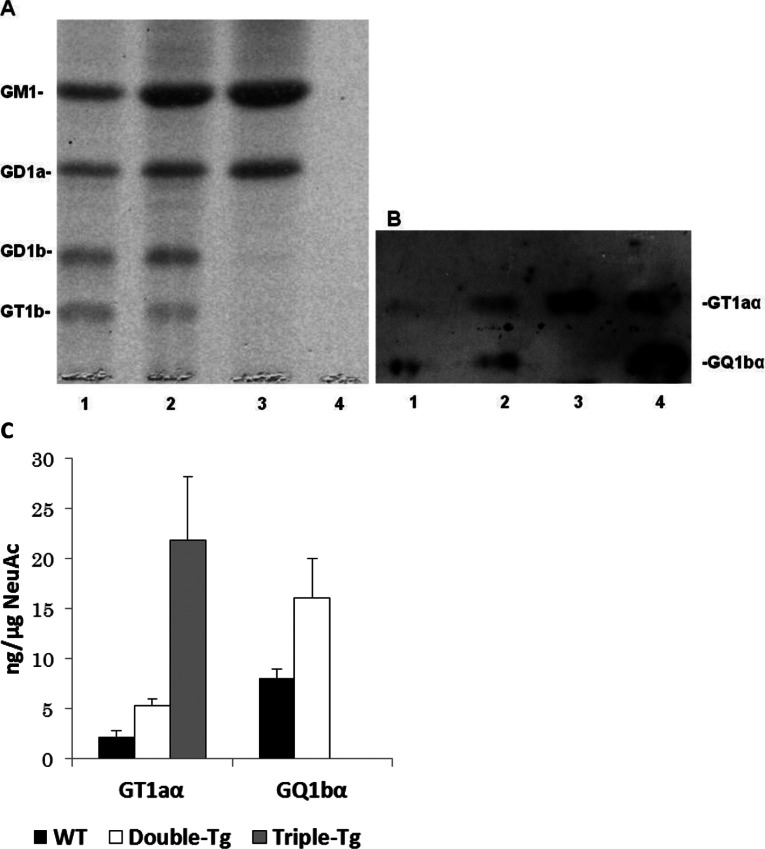
HPTLC analysis of ganglioside expression in the brains of double-mutant, triple-mutant and WT mice Gangliosides were separated by HPTLC using the solvent system of chloroform/methanol/0.2% CaCl_2_ (50:45:10, v/v) and bands were visualized by staining with the resorcinol-HCl reagent (**A**) or immunostaining with anti-Chol-1α mAb (GGR-41) (**B**). Each lane (1–3) contained 3 μg of lipid-bound sialic acid except lane 4. Lane 1, gangliosides of the brain of WT mice; lane 2, gangliosides of the brain of double-mutant mice; lane 3, gangliosides of the brain of triple-mtant mice; 4, authentic GT1aα (20 ng) and GQ1bα (20 ng), respectively. Please note that in lane 4, the amount of the two standards was too low to be revealed by the resorcinol-HCl reagent. (**C**) The concentrations of GT1aα and GQ1bα, present in the total gangliosides in each sample, were estimated by densitometric analysis of (**B**) (*n*=3). In triple-Tg mice, the concentration of GT1aα was significantly elevated compared with those in the WT and double-mutant mice. No detectable amounts of GQ1bα was present in the triple-mutant mouse brains.

GD3-synthase (STII; GD3S) is responsible for catalysing the biosynthesis of GD3, a key intermediate for the synthesis of other b- and c-series gangliosides (Figure 1). In *GD3S* gene knockout mice, all b-series gangliosides, including GD3, GD2, GD1b, GT1b and GQ1b, are deleted, and a-series gangliosides such as GM1, GD1a and GM2 show accretion (Okada et al., 2002). Chol-1α gangliosides are normally minor species in the brain and serve as unique markers of cholinergic neurons (Ando et al., 1992; Hirabayashi et al., 1992). They have also been shown immunocytochemically in human central nervous system (Obrocki and Borroni, 1988; Whittaker et al., 1992) and may find applications in human neuropathology. The expression of Chol-1α gangliosides in rat brain regions such as the hippocampus is developmentally regulated, and their concentrations increase with aging (Derrington and Borroni, 1990). Ando et al. (2004) further showed that treatment of anti-Chol-1α mAb inhibited the release of acetylcholine from synaptosomes. Interestingly, the memory and learning abilities of rats given anti-Chol-1α mAb were remarkably suppressed. On the contrary, treatment of Chol-1α gangliosides of the synaptosomal preparation induced choline uptake and enhanced acetylcholine synthesis. Thus, Chol-1α gangliosides may participate in maintaining cognitive functions such as memory and learning. In addition, Chol-1α gangliosides were shown to alleviate the decreased synaptic functions of aged brains (Ando et al., 1998). These findings suggest that Chol-1α antigens may play an important role in cholinergic synaptic transmission and participate in cognitive function. In our current study, we confirmed the expression of Chol-1α antigens in GD3S^−/−^ mice (Figure 2**A**, lane 2). As expected, there was no expression of GQ1bα in the brain of GD3S^−/−^ mice, but the levels of GT1aα were significantly elevated (Figure 2**A**, lane 2). In contrast, the concentrations of both Chol-1α ganglioside, GQ1bα and GT1aα, were increased in the brains of double-Tg mice as compared with those in the brains of WT mice, as shown in Figure 3(**B**), lane 2 and 3(**C**).

The APP/PSEN1/GD3S^−/−^ triple-mutant mice lack b-series gangliosides, which was compensated by a corresponding increase in the a-series gangliosides, GM1 (63.8%) and GD1a (50.8%), as compared with those of WT mice (Bernardo et al., 2009). In the present study, we found that the triple-mutant (APP/PSEN1/GD3S^−/−^) mice expressed GT1aα, but not GQ1bα; the lack of GQ1bα was clearly because of the deletion of the *GD3S* gene (Figure 3**C**). Remarkably, we found that deletion of the gene encoding GD3S actually resulted in a significant overexpression of GT1aα ganglioside (Figures 2 and 3).

**Figure 4:**
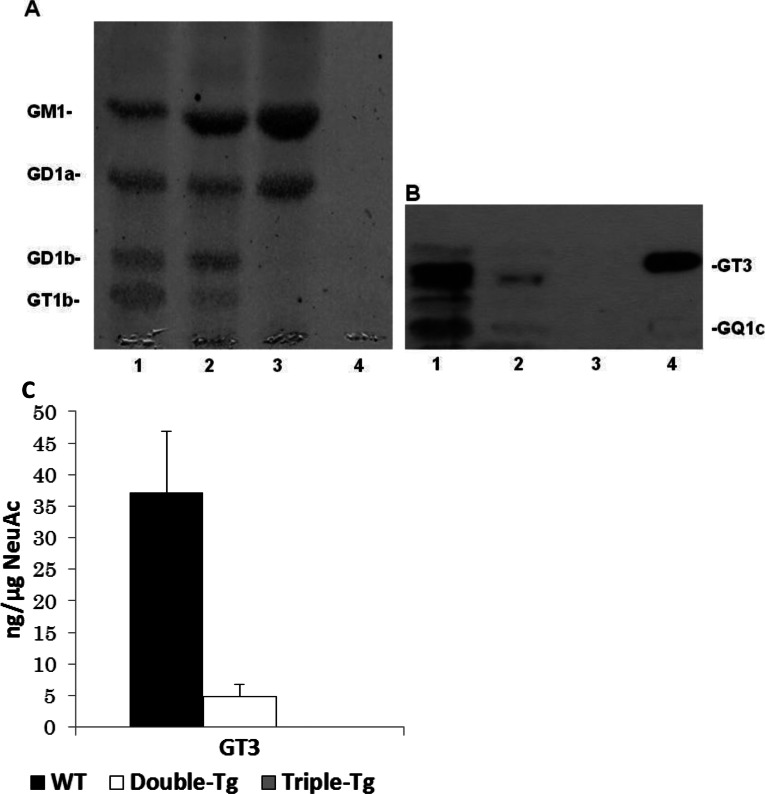
HPTLC analysis of the expression of c-series gangliosides in the brains of double-Tg, triple-Tg and WT mice Gangliosides were separated by HPTLC using the solvent system of chloroform/methanol/0.2% CaCl_2_ (50:45:10, v/v). The bands were visualized by the resorcinol-HCl reagent (**A**) or immunostaining with anti-A2B5 mAb (**B**). Each lane contained 3 μg of lipid-bound sialic acid, except lane 4. Lane 1, gangliosides in the brain of WT mice; lane 2, gangliosides in the brain of double-mutant mice; lane 3, gangliosides in the brain of triple-mutant mice; lane 4, authentic GT3 (27 ng). Please note that the amount of GT3 was too low to be detected by the resorcinol-HCl reagent. (**C**) The concentration of GT3 in the brain of WT, double-mutant and triple-mutant mice. GT3 was quantified by densitometric analysis of (**B**). *n*=3. In triple-mutant mice, there was no detectable amount of GT3.

Another interesting aspect of the ganglioside composition in the brains of double-mutant (APP/PSEN1) and triple-mutant (APP/PSEN1/GD3S^−/−^) mice was the expression of the c-series gangliosides. It is known that mouse mAb A2B5 recognizes the c-series gangliosides (GQ1c, GT1c, GT3, etc.)

(Bieberich et al., 2001; Yu et al., 2004). c-Series gangliosides are normally present in very low abundance in adult brain, but are robustly expressed in embryonic brain (Yu et al., 2004); during brain maturation the biosynthesis of gangliosides is switched from the c- to the a- and b-series gangliosides (Yu et al., 2004). For this reason, A2B5 immunoreactivity has been widely used as an indicator of immature neurons and glia. In contrast with the Chol-1α antigens, a c-series ganglioside, GT3, was significantly reduced in double-mutant (APP/PSEN1) mouse brains as compared with that in WT mouse brains (Figure 4). This finding is different from previous immunohistochemical results found in human AD brains (Takahashi et al., 1991; Tooyama et al., 1992) in which A2B5-positive antigens were found to be increased in AD brain. This discrepancy may be owing to the specificity of the mAb A2B5, which has been reported to cross-react with sulfatide in AD brains (Majocha et al., 1989). It is possible that the immunoreactivity of the mAb A2B5 used in their study could arise not only from c-series ganglioside, but also from sulfatide, resulting in an overestimation of the immunohistochemical intensity.

Recent advances on the disruption of genes encoding glycosyltransferases for ganglioside biosynthesis have facilitated studies on the functional role of these glycolipids in the pathogenesis of many neurodegenerative diseases (Yu et al., 2012). In AD studies, Oikawa et al. (2009) established Tg mice expressing human APP having Swedish and London mutations with GM2-synthase knockout mice. The mutant mice did not express GM1, GD1a, GD1b and GT1b, but robustly expressed GM3 and GD3, as reported by Takamiya et al. (1996). The mutant mice also showed a significant increase of Aβ accumulation in vascular tissues and formation of a severe dysphonic form of amyloid angiopathy in the brain. Most recently, Wu et al. (2011) reported that in mutant mice with a disrupted gene encoding GalNAcT, α-synuclein expression was significantly elevated in the substantia nigra pars compacta of the brains. These mice showed overt motor disability on aging, loss of dopaminergic neurons and aggregation of α-synuclein, resulting in Parkinson-like symptoms. On the other hand, Okada et al. (2002) established a GD3S gene knockout mouse in which all b-series gangiosides were deleted. Interestingly, these mice showed no morphological changes in the brains and apparent abnormal behaviour. Moreover, no differences in Fas-mediated apoptotic reaction in lymphocytes compared with the WT mice were found. However, the mutant mice exhibited reduced regeneration of axotomized hypoglossal nerves compared with the WT mice, suggesting that b-series gangliosides are more important in the repair of damaged nerves than in the differentiation of the nervous system. In our previous study, we (Bernardo et al., 2009) established the triple-mutant mouse model of AD (APP/PSEN1/GD3S^−/−^) in which all b-series gangliosides, including GD3, were absent, but GM1 and GD1a were significantly increased. Interestingly, Aβ plaques and the associated neuropathology were almost completely absent in the triple-mutant mice, which showed no impairment of cognitive functions. These observations suggest that b-series gangliosides derived from the action of GD3S are one of the major causes of Aβ accumulation and AD. Analysis of these triple-mutant mice may contribute to our understanding of the neurobiological and behavioural characteristics of Aβ-ganglioside interaction as an essential step toward elucidating the early pathological events in AD. Thus, inhibition of GD3S may be a useful therapeutic target to restore the cognitive deficits, amyloid plaque formation, and neurodegeneration observed in AD (Bernardo et al., 2009). Several investigators have described aberrant ganglioside metabolism may be participated in the pathogenesis in AD (for review, see Ariga et al., 2011). In this regard, it is interesting to note that chronic infusion of GM1 into the lateral ventricle of patients with AD for 1 year resulted in remarkable improvement of their memory function (Svennerholm et al., 2002). The underlying mechanism of the GM1-induced effect is a subject of considerable speculation. Nonetheless, an increased level of GM1 in the triple mutant mice is consistent with the above observation and may also contribute to the observed improved cognitive improvement in these mice.

In conclusion, Chol-1α antigens and A2B5-positive c-series gangliosides are normally found as minor components in mouse brains and their expression is developmentally regulated (Ngamukote et al., 2007). At present, the functional role of those gangliosides, especially Chol-1α, in the pathogenesis of AD is still unknown. Our current findings that those gangliosides are overexpressed in the triple Tg model of AD, which also exhibits improved memory function and no amyloid formation, could provide some important directions for future research.
